# 98 Health care practitioners’ knowledge, use and confidence of PA guidelines and tools during pregnancy on the island of Ireland: a protocol and preliminary data

**DOI:** 10.1093/eurpub/ckae114.170

**Published:** 2024-09-26

**Authors:** Elizabeth Deery, Maria Faulkner, Sinead Currie, Ben Fitzpatrick

**Affiliations:** Ulster University, Derry/Londonderry, Northern Ireland; Atlantic Technological University, Letterkenny, Ireland; University of Stirling, Stirling, Scotland; Ulster University, Derry/Londonderry, Northern Ireland

## Abstract

**Purpose:**

Regular physical activity (PA) during pregnancy is associated with benefits to maternal and foetal health, and the World Health Organisation recommend 150 minutes of weekly PA during pregnancy. Despite this, PA participation often decreases during pregnancy, and this may negatively impact mother-child PA participation later in life. Healthcare practitioners (HCPs) are considered well-placed to deliver health-behaviour messaging; however, little is known about the delivery of this in a maternity context. This study therefore aims to evaluate the delivery of PA guidance by HCPs during pregnancy, their knowledge of PA guidelines and resources, and their confidence in implementing these, across the island of Ireland.

**Methods:**

Ethical approval will be sought from Ulster University research ethics committee. From June to September 2024, participants, who will be HCP involved in maternity care on the island of Ireland, will be invited to take part in an anonymous, online survey. Invitations will be circulated via professional bodies, networks, social media, and snowball sampling. The survey, which will be developed in conjunction with an expert advisory panel, will assess HCP knowledge, confidence, and delivery of PA guidelines and tools during pregnancy, and assess training received on PA guidance and PA tools during pregnancy. Statistical analysis will include independent t-tests, chi-squared, Pearson correlation, frequencies, and descriptive data. Content analysis will be used to analyse data produced from open-ended questions.

**Results:**

Preliminary findings will include participant demographics and descriptive data regarding the knowledge, confidence, training in and prevalence of delivering PA guidance and PA tools during pregnancy. Statistical analysis will be subject to sample-size achievement at this preliminary stage.

**Conclusions:**

The findings from this study have the potential to identify gaps in the knowledge and confidence of HCP delivering PA guidance during pregnancy. This will add knowledge to the extant literature and can provide a foundation to inform interventions and strategies aimed at enhancing the delivery of evidence-based practice regarding PA during pregnancy. This would have the potential to improve the quality of prenatal care, promote maternal and fetal health, and enhance health promoting health-enhancing PA in the prenatal population.

**Funding Source:**

No funding to declare.

